# Assessment of the Liver Steatosis and Fibrosis Risk in Metabolic Syndrome and Its Individual Components, Considering the Varying Definitions Used in Clinical Practice throughout Time: A Retrospective Cross-Sectional Study

**DOI:** 10.3390/biomedicines12081739

**Published:** 2024-08-02

**Authors:** Szymon Suwała, Roman Junik

**Affiliations:** Department of Endocrinology and Diabetology, Nicolaus Copernicus University, Collegium Medicum, 9 Sklodowskiej-Curie Street, 85-094 Bydgoszcz, Poland; junik@cm.umk.pl

**Keywords:** liver steatosis, hepatofibrosis, metabolic syndrome, diabetes, diabesity, obesity

## Abstract

Multiple modifications of metabolic syndrome diagnostic criteria have been made—NCEP: ATP III (from 2001, modified in 2004), IDF (2005), IDF Consortium (2009), or Polish Scientific Society Consortium standards (2022) are now frequently in use. Hepatosteatosis and hepatofibrosis are commonly mentioned aspects of metabolic syndrome that greatly increase the likelihood of developing complications. The objective of the study was to assess different diagnostic criteria for metabolic syndrome based on the prevalence of liver steatosis and fibrosis. A retrospective analysis was conducted on the medical data of 2102 patients. Out of all the single criteria, meeting the obesity criterion based on waist circumference showed the highest increase in the risk of hepatosteatosis (by 64–69%, depending on the definition used)—hypertriglyceridemia increased the risk of hepatofibrosis by 71%. Regardless of the specific criteria used, patients with metabolic syndrome had a 34–36% increased likelihood of developing hepatosteatosis—the probability of hepatofibrosis varied between 42% and 47% for the criteria established in 2004, 2005, and 2009, while the Polish 2022 criteria were not statistically significant (*p* = 0.818). It seems appropriate to establish consistent metabolic syndrome diagnostic criteria—the 2009 IDF guidelines are the most effective in assessing hepatosteatosis and fibrosis risk.

## 1. Introduction

Metabolic syndrome is a widespread worldwide health and societal problem marked by an increased likelihood of cardiovascular events due to the simultaneous existence of obesity and other variables, such as hypertension or disorders of glucose and lipid metabolism. However, various definitions are used to define metabolic syndrome in adults. From a historical perspective, Reaven introduced the concept of syndrome X (later called “metabolic syndrome”) in 1988, asserting that insulin resistance is the primary feature that contributes to the emergence of cardiovascular diseases and type 2 diabetes, both of which are part of the aforementioned X syndrome [1]. Since then, many scientific societies worldwide have taken part in attempts to define metabolic syndrome, for example the World Health Organization (WHO), the European Group for the Study of Insulin Resistance (EGIR), the National Cholesterol Education Program Adult Treatment Panel III (NCEP: ATPIII), the American Association of Clinical Endocrinology (AACE), the International Diabetes Federation (IDF, which later collaborated with other societies such as the American Heart Association: AHA or the National Heart, Lung, and Blood Institute: NHLBI), and, in 2022, also a consortium of Polish scientific societies [2,3,4,5,6,7]. Even a cursory review of the scientific literature reveals that the NCEP: ATP III, the IDF from 2005, and the IDF consortium from 2009 definitions of metabolic syndrome are those that are most frequently used in clinical settings—in Poland, the home country of the authors of this manuscript, highly appreciated are also Polish experts guidelines from 2022. The differences between them (Table 1 presents a comparison of these four definitions for the Caucasian population), although seemingly subtle, de facto revolved mainly around the varying degrees of influence of obesity and/or abnormalities in carbohydrate metabolism, each defined differently by different authors [8]. All of this leads to significant confusion, particularly when considering the significance of diagnosing metabolic syndrome, a condition in which interconnected metabolic variables have a substantial impact on cardiovascular risk and are related to mortality.

One of the components and complications of the metabolic syndrome is liver dysfunction, specifically steatosis and fibrosis, which are considered important hepatic factors in the metabolic syndrome. These conditions are seen as sensitive indicators of an increased risk of cardiovascular events, as mentioned in scientific articles [9,10,11,12,13,14]. In this case, it is reasonable to determine which diagnostic criteria for the metabolic syndrome, if met, indicate the highest risk of the observed abnormalities in liver structure.

This study aimed to evaluate the risk of liver steatosis and advanced hepatofibrosis in groups of patients who fulfilled various metabolic syndrome criteria (from 2004, 2005, 2009, and Poland from 2022), with consideration also given to the risk of each criterion separately.

## 2. Materials and Methods

The authors analyzed retrospective medical data from patients in their endocrinology department who were hospitalized between 2013 and 2020 (before the first cases of COVID-19 appeared in Poland, which was related to reports appearing in the literature on the potential impact of SARS-CoV2 virus infection on liver health [15,16,17]). The analysis included data from patients aged 18–65 who were admitted to the hospital and in whom all necessary factors were evaluated: weight, height (to calculate body max index—BMI), waist circumference, blood pressure, fasting glucose, glycated hemoglobin, lipid profile (all of the above to assess each component of the metabolic syndrome), alanine and aspartate aminotransferases (ALT, AST), platelet count (to calculate FIB-4 for hepatofibrosis prediction), and liver ultrasonography or magnetic resonance imaging (to assess hepatosteatosis). The study considered data only from patients who were admitted to the hospital for the diagnosis of incidentally detected pituitary, adrenal, and thyroid tumors (in whom diagnostic hospitalization confirmed that these lesions were silent and hormonally inactive), as well as patients diagnosed with obesity (only those in whom the hormonal cause of obesity, such as Cushing’s syndrome, had not been confirmed) and who were admitted for diabetes (re)education and therapy optimization. The authors excluded documentation from patients who were hospitalized with any acute internal medicine disorders (like pneumonia, urinary tract infections, sepsis, anemia, exacerbation of chronic kidney failure or heart failure, and others), life-threatening conditions, and from any patients with alcohol dependence syndrome (in history or current). Finally, data from a total of 2102 unique patients was finally included: 1247 women (59.32%) and 855 men (40.68%).

The study assessed whether hospitalized patients fulfilled the particular criteria for the metabolic syndrome (as outlined in Table 1); each criterion was assigned a unique abbreviation (as presented in Table 2) for later easier description—we chose not to analyze the criterion of hyperglycemia associated with the oral glucose tolerance test (because, according to the internal hospital guidelines, this test was only performed when fasting glucose exceeded 100 mg/dL, which was already met by another criterion, HG1) and the criterion of hypertension based on the patient’s home measures (due to data unavailability—authors include only information about measures done in the hospital).

In the next step, we evaluated the prevalence of liver steatosis in radiological examination reports and calculated the FIB-4 index for liver fibrosis prediction (according to the following formula: (age [years] × AST [U/L])/(platelet [10^9^/L] × √ALT [U/L]), like proposed in the original publication of inventors [18]) for those who fulfilled specific criteria (both individually and collectively—for the metabolic syndrome in general). To determine suitable thresholds for identifying a higher risk of advanced liver fibrosis, the available literature was examined—it was determined that the recommendations of Ishiba et al. (categorizing the FIB-4 index according to the age of patients) would be utilized: ≥1.21 for patients up to 49 years of age (1081 individuals), ≥1.96 for patients aged 50–59 years (587 individuals), and ≥2.67 for patients aged 60–69 years (434 subjects) [19,20].

The acquired results were analyzed statistically using STATISTICA 13.0 PL statistical software (TIBCO Software Inc., Palo Alto, CA, USA). Mean and standard deviation (SD) are used to represent continuous variables (results of laboratory parameters), whereas exact numbers and percentages are used to represent categorical variables. Evaluation of relative risks (RR) was employed using the Chi-squared test, each time also providing the 95% confidence interval (95%CI). The threshold for statistical significance was established at 0.05.

## 3. Results

### 3.1. General Characteristics of Patients

An analysis was conducted on the medical records of 2102 patients, primarily consisting of women (1247 individuals; 59.32%)—the average age of the patients was 46.10 ± 14.13 years and did not differ significantly among genders (*p* = 0.347). The mean BMI was 31.63 ± 4.68 kg/m^2^ (with no difference between men and women; *p* = 0.166). The waist circumference was 102.64 ± 11.41 cm (in the male group) and 91.62 ± 9.93 cm (in the female group)—the difference was statistically significant (*p* < 0.001). The average systolic and diastolic blood pressure was 134.00 ± 10.63 and 80.13 ± 6.65 mmHg, respectively (with no statistically significant difference between men and women; *p* = 0.132). Table 3 presents the mean values of laboratory measures required to evaluate specific criteria of the metabolic syndrome and the age-dependent risk of hepatofibrosis.

**Figure 1:**
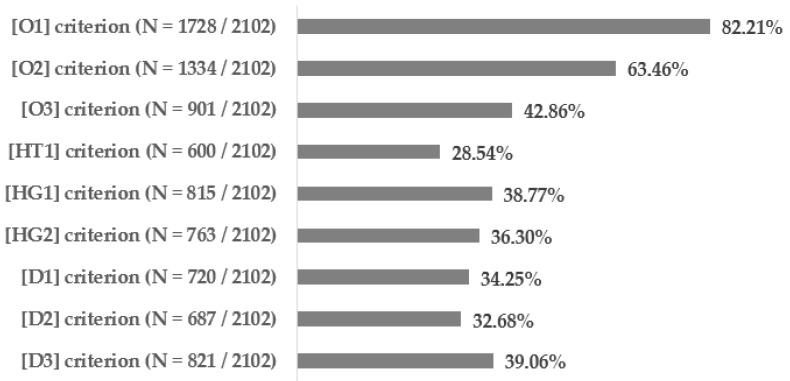
Percentages of patients who meet each criterion of metabolic syndromes.

Figure 1 illustrates the percentages and exact numbers of patients who meet each specific requirement for defining metabolic syndromes (described in Table 2). The proportions of study participants who fulfilled the combined criteria for the diagnosis of metabolic syndrome were as follows: 34.02% (715) according to the NCEP: ATP III criteria from 2004, 37.73% (793) based on the IDF criteria from 2005, 38.34% (806) according to the IDF consortium criteria from 2009, and 24.82% (521) in accordance with the Polish experts consensus from 2022.

Liver steatosis was observed in 64.89% (1364) of all patients, and features of increased risk of advanced hepatofibrosis (based on the age-dependent FIB-4 index) were present in 218 patients (10.37%).

### 3.2. Risk of Hepatosteatosis and Hepatofibrosis Assessed by Compound Criteria of Metabolic Syndromes

**Figure 2:**
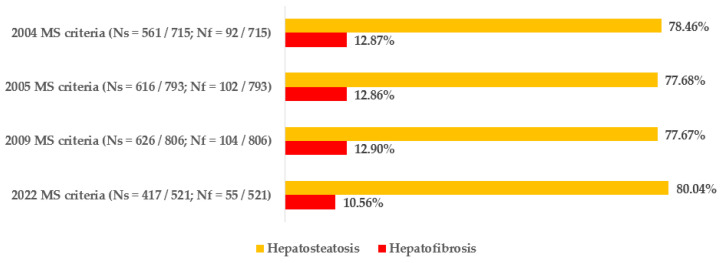
Prevalence of hepatosteatosis and hepatofibrosis (assessed by FIB-4 index) in groups of patients who meet specific compound criteria for metabolic syndrome (MS).

Figure 2 displays the prevalence of liver steatosis (exact number: Ns) and fibrosis (exact number: Nf) among groups of patients who meet specific criteria for the diagnosis of metabolic syndrome. Steatosis was seen in 77.67–80.04% of them (depending on certain criteria). The FIB-4 index showed that fibrotic features were present in 10.56–12.90% of people with metabolic syndrome.

**Figure 3:**
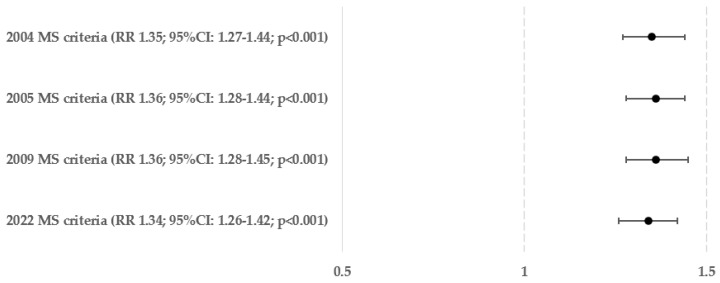
Relative risk of hepatosteatosis in compound criteria of metabolic syndromes (MS).

**Figure 4:**
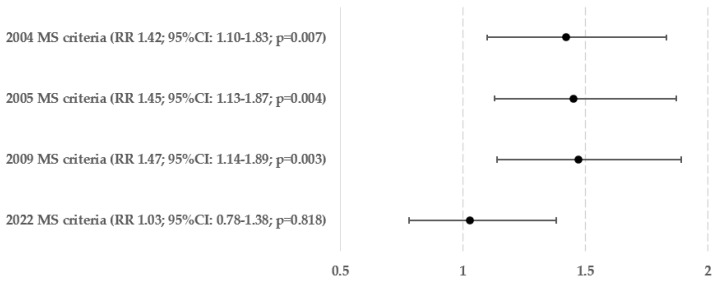
Relative risk of hepatofibrosis in compound criteria of metabolic syndromes (MS).

The risks of hepatosteatosis and advanced hepatofibrosis in people who meet the compound criteria for metabolic syndrome from 2004, 2005, 2009, or 2022 are shown in Figure 3 and Figure 4. In the context of fatty liver, the risk of all criteria was nearly identical (being higher by 34–36%). However, in the case of liver fibrosis, it was observed that the Polish criteria did not demonstrate statistical significance (*p* = 0.818). The probability of hepatofibrosis in patients who met the criteria in 2004, 2005, and 2009 was 42–47% greater (the most for the group with fulfilled IDF consortium criteria from 2009).

### 3.3. Risk of Hepatosteatosis and Hepatofibrosis Assessed by Single Criterion of Metabolic Syndrome

**Figure 5:**
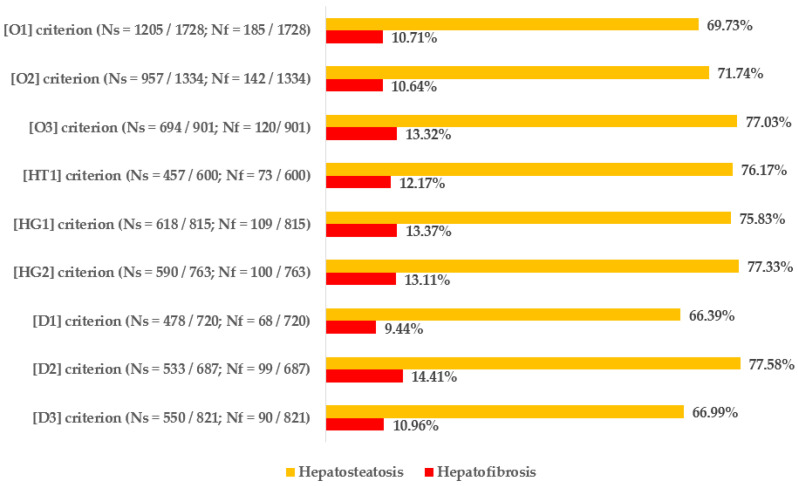
Prevalence of hepatosteatosis and hepatofibrosis (assessed by FIB-4 index) in groups of patients who meet every criterion of metabolic syndrome.

**Figure 6:**
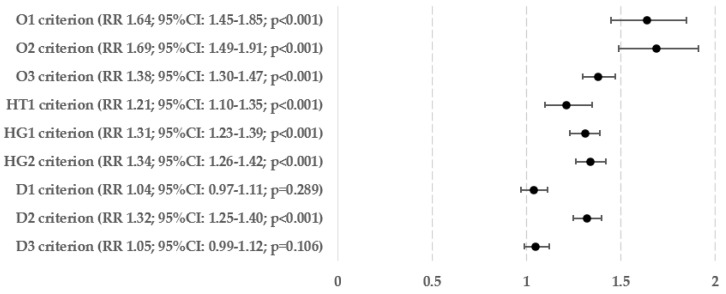
Relative risk of hepatosteatosis in every single criterion of metabolic syndromes.

**Figure 7:**
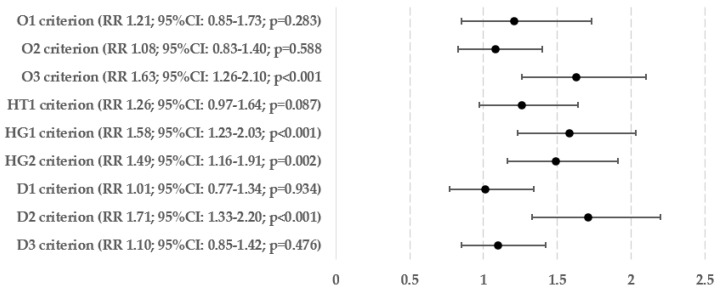
Relative risk of hepatofibrosis in every single criterion of metabolic syndromes.

Figure 5 displays the percentages and exact numbers of patients who met a specific single criterion of metabolic syndrome and have been diagnosed with hepatic steatosis (Ns) and those who are at risk of developing advanced liver fibrosis (Nf). Figure 6 and Figure 7 show the relative risks of hepatic steatosis and the chance of advanced hepatofibrosis compared to those who do not meet this metabolic syndrome criterion.

The highest incidence of liver steatosis was observed in patients meeting the criterion of hypertriglyceridemia (77.58%), carbohydrate metabolism disorders based on HbA1c percentage (77.33%), and obesitological criterion based on BMI (77.03%). Regarding liver fibrosis, the highest percentage of high chance of developing advanced hepatofibrosis was observed in patients meeting the criterion of hypertriglyceridemia (14.41%), hyperglycemia (13.37%) and obesity based on BMI (13.32%).

In relation to the relative risk of hepatosteatosis, fulfilling any of the criteria related to obesity raises the risk by 38–69%—meeting the criterion for hypertension increases the risk by 21%, and fulfilling any of the requirements for (pre)diabetes increases the risk by 31–34%. Hypertriglyceridemia, which raised the risk of steatosis by 32%, was the only factor that showed a statistically significant link with lipid metabolism disorders. The likelihood of having an above-normal FIB-4 score, which is quite accurate in predicting advanced liver fibrosis, was significantly higher in patients who met the criteria for obesity based on BMI (by 63%), both criteria for carbohydrate metabolism disorders (by 49–58%), and the criterion for hypertriglyceridemia (by 71%).

### 3.4. Risk of Hepatosteatosis and Hepatofibrosis Assessed by Criteria Within the Same Clinical Group

**Figure 8:**
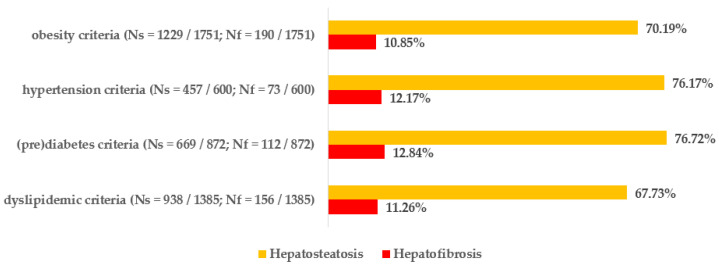
Prevalence of hepatosteatosis and hepatofibrosis (assessed by FIB-4 index) in groups of patients who meet any criterion from the same clinical group.

**Figure 9:**
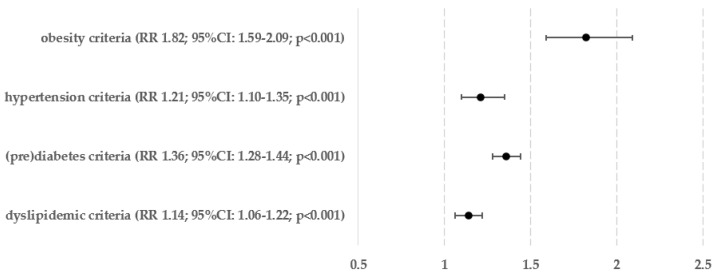
Relative risk of hepatosteatosis in groups of patients who meet any criterion from the same clinical group.

**Figure 10:**
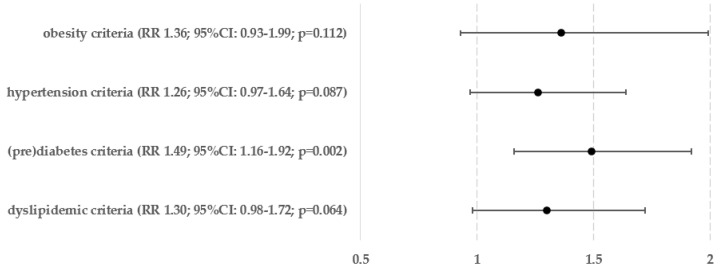
Relative risk of hepatofibrosis in groups of patients who meet any criterion from the same clinical group.

An attempt was made to evaluate the frequency and relative risks of liver steatosis and fibrosis in individuals who meet any of the specified criteria from a particular group (including obesity-related, hypertensive, carbohydrate metabolism disorders, and lipid metabolism disorders), i.e., O1 or O3 (O2 as a narrower criterion is already included in O1; 1751 patients meeting the criteria: 83.30%); HT1 (as the sole hypertension criterion investigated in the study); HG1 or HG2 (872 patients: 41.48%); and D1, D2, or D3 (1383 patients: 65.89%). As with the previously shown figures, the data are shown in the next ones, labeled with integers ranging from Figure 8, Figure 9 and Figure 10.

Meeting any of the obesity criteria was the main risk factor for hepatosteatosis, resulting in an 82% increase in risk. Subsequently, the presence of carbohydrate metabolism disorders raised the risk by 36%, whereas hypertension, as previously stated, increased the risk by 21%, and dyslipidemics by 14%. When examining advanced hepatofibrosis, the only group that exhibited a statistically significant rise in relative risk was the one associated with abnormalities in glucose metabolism, showing a 49% increase.

## 4. Discussion

Multiple definitions are employed to identify metabolic syndrome in adult individuals. This cross-sectional study aimed to compare the usual criteria and the newest criteria developed by Polish scientific societies for assessing the risk of liver steatosis, which refers to abnormalities in the structure and function of the liver that are associated with an elevated risk of cardiovascular events. Mellinger et al. confirmed that liver steatosis was clinically significantly associated with the presence of coronary and abdominal artery calcium [21]. Two prospective studies conducted by Fracanzani et al. and Baratta et al. show that liver steatosis is independently linked to a 2–2.5 times increased risk of cardiovascular events [22,23]. Hepatosteatosis can progress, leading to the development of steatohepatitis and, finally, fibrosis, which manifests in around 50% of patients over a span of 8 to 13 years [24]. Perazzo et al., from 2014, the presence of advanced fibrosis in a group of patients with a Framingham-risk score ≥ 20% was predictive of cardiovascular events (HR 2.24; 95%CI: 1.16–4.33) [25].

Our investigation revealed that patients who matched all “classic” criteria for the metabolic syndrome, as defined in the years 2004, 2005, and 2009, had a higher relative risk of developing both hepatic steatosis and fibrosis. Regarding the NCEP: ATP III criteria, the risk was found to be 35% and 42% higher, respectively. When using the IDF criteria from 2005—36% and 45% higher, and when considering the IDF consortium criteria in conjunction with other societies from 2009, the risk was 36% and 47% higher—and the latter criteria should be regarded as the most effective in identifying patients who are at an elevated risk of liver structural abnormalities. Despite the popularity of scientific articles discussing liver steatosis (including non-alcoholic fatty liver disease: NAFLD and metabolic associated fatty liver disease: MAFLD) and metabolic syndrome, it is challenging to find studies that examine the simultaneous occurrence and risk of these disorders—especially when considering additionally the risk of liver fibrosis and the use of the latest diagnostic criteria for metabolic syndrome from Poland (which is understandable given their recent introduction). Regarding the Polish criteria, patients who meet these criteria for diagnosing metabolic syndrome have a 34% increased risk of developing hepatosteatosis. However, the assessment of the relative risk of liver fibrosis yielded statistically insignificant results. It should be noted that the Polish criteria for diagnosing the metabolic syndrome differ significantly from the “classic” definitions. In addition to the required component related to obesity (which is also present in the IDF criteria from 2005, but without considering BMI), there is a distinct criterion related to dyslipidemia (focused on the concentration of non-HDL cholesterol instead of considering the concentrations of HDL cholesterol and triglycerides) and a new optional criterion for hyperglycemia (percentage of HbA1c) [7]. Although these criteria may effectively evaluate cardiovascular risk, we cannot endorse their use for assessing the risk of liver structural abnormalities (especially advanced, like hepatofibrosis).

The metabolic syndrome includes four main components, irrespective of the chosen definition, which pertain to obesity, hypertension, carbohydrate metabolism disorders, and abnormalities in lipid metabolism. Two of the four discussed compound criteria (IDF from 2005 and Polish consensus from 2022) treat obesity as an absolute condition for diagnosing metabolic syndrome, considering the other criteria as supplementary factors [6,7]—this strategy has its basis in studies that suggest obesity is not just an independent risk factor for cardiovascular events but also the most important modifiable factor that contributes to other above mentioned health conditions [26,27,28]. Our study revealed that in the context of liver steatosis, the obesity criteria actually presented the highest relative risk (with an increase of 38–69%), but in the case of liver fibrosis, only the criterion based on BMI was statistically significant, resulting in a 63% higher risk—additionally, hypertriglyceridemia was found to be superior, increasing the risk by 71%. Hepatocytes accumulate triglycerides due to an increased influx of free fatty acids and enhanced beta-oxidation—however, when mitochondrial beta-oxidation of fatty acids fails, it triggers the expression of the 2E1 isoform of cytochrome P450 (CYP2E1). This, in turn, intensifies oxidative stress in the liver, initiating inflammation and fibrosis and reduces the hepatic synthesis of apolipoprotein B-100 and very low-density lipoproteins (VLDL). It becomes more intriguing when we consider our risk assessment in relation to meeting any specific criteria in certain clinical groups (obesity, hypertension, carbohydrate or lipid metabolism disorders)—when assessing the risk of steatosis, obesity was again found to be the main factor increasing the risk (by 82%), but on the other hand, for fibrosis, the only factors with significantly higher relative risk were prediabetes and diabetes disorders (an increase of 49%). It is important, however, to note that insulin resistance (due, for example, to obesity) is probably the main underlying cause of this entire process [29,30].

When mentioning obesity, it is worth paying attention to the differences in the assessment of the risk of liver structure disorders depending on the adopted criterion for diagnosing obesity, which is based on waist circumference or BMI. In our study, the incidence of both hepatic steatosis and fibrosis was higher in patients meeting the BMI-based obesity criterion than either waist circumference-based criterion (77.03% > 71.74% > 69.73% for fatty liver and 13.32% > 10.64% > 10.71% for hepatofibrosis). These results are slightly different from those previously published. A study conducted by Rocha et al. found that liver fibrosis confirmed by biopsy (in the course of non-alcoholic steatohepatitis: NASH) occurred in 9 out of 34 patients (26.45%) with an increased waist circumference and in 6 out of 32 patients (18.75%) with a BMI ≥ 30 kg/m^2^—however, it is important to mention that the liver biopsy in this study was only conducted in 37 out of the 81 patients [31]. In 2021, Chinese researchers developed a decision tree model that accurately estimates the prevalence of NAFLD in the adult population—the structure of this decision tree is primarily based on waist circumference, which is identified as the most significant attribute (BMI was completely disregarded in this model) [32]. Hamaguchi et al. in their study, they did not find a significant difference between obesity defined by BMI and waist circumference in the incidence of NAFLD [33]—it should be remembered, however, that the cut-off points for diagnosing abdominal obesity based on both BMI and waist circumference in the Asian population are different. The analysis of data published by Claypool et al. reveals that in individuals with a BMI indicating obesity, the prevalence of liver steatosis and fibrosis is approximately 45% and 16%, respectively, and in patients with an increased waist circumference, the rates are approximately 40% and 13% (in both cases, definitions were used after accounting for population differences)—these findings are somewhat in line with our own results [34]. The equivocal and inconsistent findings prompt us to consider the necessity of conducting additional studies in this field. Upon analyzing our risk data, it can be noticed that the criteria based on waist circumference exhibit greater significance in relation to hepatosteatosis (the risk of developing this problem increased by 64–69% when considering waist circumference and by 38% when considering BMI)—regarding fibrosis, however, only obesity defined by BMI exhibited a statistically meaningful outcome, with a 63% increase in the probability of advanced hepatofibrosis. A comprehensive examination of all criteria related to obesity clearly shows that, unlike liver steatosis, in the risk assessment of liver fibrosis, definitions of obesity based on waist circumference and BMI cannot be used interchangeably at the same time (*p* = 0.112). In conclusion, our findings suggest the need to tailor the definition of obesity based on the specific goal of assessment: for the evaluation of hepatic steatosis risk, waist circumference appears to be a more suitable parameter, whereas BMI is more appropriate for assessing the risk of advanced liver fibrosis.

One of the other components of the metabolic syndrome with interesting observations in the results of our study are the indicators of hyperglycemia: fasting glucose and percentage of glycated hemoglobin. These criteria are the only factors that exhibit a statistically and clinically significant relative risk of both fatty liver and hepatofibrosis, regardless of their specific definitions. It is important to note again that we did not analyze the glycemia after 120 min of the 75-g oral glucose tolerance test as one of the hyperglycemic criteria because our hospital and department guidelines state that this test is only performed in patients who have already been diagnosed with fasting hyperglycemia, which fulfills another criterion of hyperglycemia. Regardless of whether the hyperglycemic component of the metabolic syndrome is referred to as fasting hyperglycemia or as an elevated percentage of glycated hemoglobin, patients who meet this criterion have a 31–34% higher risk of developing steatosis (with an advantage for the HbA1c criterion) and a 49–58% higher risk of hepatofibrosis (with a greater predominance for fasting hyperglycemia). Many previous studies have confirmed that patients with (pre)diabetes are more predisposed to the development of fatty liver and hepatofibrosis [35,36,37,38,39]—as Ciardullo describes, 15–38% of patients may have even advanced features of liver fibrosis, including cirrhosis (stages F3–F4) [40]. In this regard, we are completely consistent; however, our study seems to be the first to evaluate two separate definitions of carbohydrate metabolism disorders to investigate two distinct definitions of carbohydrate metabolism disorders in relation to heightened metabolic risk while also considering the prevalence of liver structural problems.

The presence of liver steatosis in a patient with type 2 diabetes, as well as in a patient with obesity, fulfills the criteria for diagnosing MAFLD [41]. The simultaneous presence of these two diseases (where insulin resistance is a common link between both) may be called diabesity—if, in this condition, there are indications of fibrosis identified through non-invasive markers, imaging tests, transient elastography, or a liver biopsy, we can classify it as diabesity-related fibrosis. The only work published on this subject so far demonstrates that diabesity is distinguished by a 2.8-fold elevated likelihood of liver fibrosis, and this risk escalates with each level of obesity as measured by BMI, reaching 3.65 times the risk in individuals classified as having obesity degree III (BMI > 40 kg/m^2^) [42]. Given the significant impact of obesity on the complications of metabolic syndrome, as previously demonstrated in many studies, and our observations regarding carbohydrate metabolism disorders and diabesity (especially in the context of liver function), it is worth considering diabesity as a major contributor to metabolic complications. Future research should focus on investigating this hypothesis and identifying the most effective strategies for prevention, diagnosis, and treatment of diabesity. A potential clue in this last issue seems to be drugs used in the treatment of diabetes, obesity, and cardiovascular diseases, specifically GLP-1 analogs and/or SGLT-2 inhibitors [43]. Obesity and diabetes (and prediabetes) are classified as endocrine disorders clearly related to the etiopathogenesis of liver steatosis, and it is recommended to undergo periodic ultrasonography abdomen examinations (or transient elastography) following diagnosis at least once every year [44].

A noteworthy finding is that individuals who met the criteria for hypertension did not exhibit a statistically significant risk of advanced hepatofibrosis, although they did have a 21% higher risk of developing fatty liver disease. The observed association between fatty liver disease and risk is consistent with other scientific research findings that have established a connection between these two conditions [12,45,46,47,48]. Nevertheless, there is a lack of consensus on the association between liver fibrosis and hypertension. While most studies consider hypertension as a risk factor for the development and advancement of hepatofibrosis [49,50,51], the research conducted by Aneni et al. suggests that hypertension may actually have a protective effect against the progression of severe liver structural abnormalities [52]. However, this work was based only on the FIB-4 index (also standard, not age-dependent), which, although quite sensitive, is not ideal—and perhaps this is a limitation of both the mentioned and our work.

Although our study is the first to evaluate the risk of hepatic steatosis and advanced fibrosis based on different criteria for metabolic syndrome in patients (developed by NCEP: ATP III, IDF, or Polish experts), it is important to note that similar efforts have been made previously—for instance, Schreiner et al. in 2021 demonstrated associations between elevated FIB-4 and NFS scores and low HDL-C and hyperglycemia features (however, it is worth mentioning that due to the unavailability of certain data in a retrospective analysis, the researchers used substitutes for the classic components of metabolic syndrome, such as HbA1c percentage instead of fasting glucose and waist circumference instead of BMI—this limitation was acknowledged and emphasized by the authors) [53].

This work has its limitations. The primary limitation of this study is that it is a retrospective cross-sectional study based solely on the analysis of data from medical records without the ability to verify them in real-time patient situations. Consequently, certain valuable information, such as smoking habits, alcohol consumption, and specific details regarding diet or physical activity, was incomplete or unavailable to the authors during data collection. The population of patients hospitalized in the endocrinology and diabetology department for diagnostic, optimization, or re-educational purposes may also potentially differ from the base population. Another possible limitation is that we relied only on a non-invasive predictive marker instead of objective test data in our analysis of liver fibrosis—it is important to mention that the current gold standard for diagnosing hepatofibrosis is invasive liver biopsy; however, FIB-4, especially age-dependent, demonstrates high sensitivity and predictive accuracy [20]. Scientists are increasingly challenging the notion of a “gold standard” in liver biopsy, not due to the lack of accuracy in its results but rather because of the high cost and potential risks associated with the procedure—they propose using non-invasive methods (such as the FIB-4 test or elastography like Fibroscan) as a means to reduce this risk [54]. Our team intends to carry out a prospective study in the future, based on the general population, using data from liver elastography tests and age-dependent FIB-4, NAFLD Fibrosis Score or LFRI (Liver Fibrosis Risk Index, which showed high sensitivity in risk stratification of MAFLD patients and may be a promising alternative to invasive liver biopsy [55])—this approach will greatly enhance the quality of the obtained results. Cooperation with a center that routinely performs liver biopsy is also being considered to use this gold diagnostic standard in the research results. Anyway, we are optimistic that this publication will serve as a source of inspiration for other scholars in this field, regardless of its limits.

## 5. Conclusions

Liver steatosis and fibrosis are significant elements or complications of metabolic syndrome. Depending on the specific definition employed, the diagnosis of metabolic syndrome is associated with a 34–36% increased risk of steatosis and a 42–47% increased risk of fibrosis (with the exclusion of the latest guidelines for diagnosing metabolic syndrome from Poland, which do not permit a statistically significant assessment of the risk of liver fibrosis). To identify patients with the greatest susceptibility to these problems, it is advisable to employ the definition established by the International Diabetes Foundation consortium in collaboration with other scientific associations in 2009. Obesity is the most significant risk factor for hepatosteatosis, particularly when defined by increased waist circumference—but on the other hand, hypertriglyceridemia is the strongest risk factor for hepatofibrosis (although obesity is also a significant risk factor for liver fibrosis, it is only defined by the body mass index, unlike steatosis). Carbohydrate metabolism disorders are the only category of illnesses in the metabolic syndrome that consistently have a higher risk of both hepatic steatosis and fibrosis, regardless of how we define them. Considering the aforementioned, it is important to note that when assessing the risk of liver structural disorders, the concept of diabesity (the simultaneous occurrence of diabetes and obesity) and the prediction indicators for these disorders proposed in publications from recent years based on the concentration of triglycerides, glucose, and anthropometric indicators should be taken into account.

This study is the first assessment of the diagnostic criteria for metabolic syndrome developed by NCEP: ATP III, IDF, and Polish experts with regard to the risk of hepatic steatosis and advanced fibrosis, including breaking down the entire sets into their components. However, further research is required to broaden our understanding of this field. For now, practitioners should consider the findings of this study when actively identifying individuals who are at risk of liver structural abnormalities (and, secondarily, functional liver issues and cardiovascular events).

## Figures and Tables

**Table 1:** Comparison of metabolic syndrome criteria from 2004, 2005, 2009, and 2022 in the Caucasian population.

Component	NCEP: ATP III Criteria (2004)	IDF Criteria (2005)	IDF Consortium Criteria (2009)	Polish Experts Consensus Criteria (2022)
**Obesity component**	waist circumference ≥88 cm (females) or ≥102 cm (males)	waist circumference ≥80 cm (females) or ≥94 cm (males)		waist circumference ≥88 cm (females) or ≥102 cm (males); or BMI ≥ 30 kg/m^2^
**Hypertension** **component**	≥130/85 mmHg or hypertension treatment			≥130/85 mmHg (measured in clinic) or ≥130/80 mmHg (measured by patient); or hypertension treatment
**Hyperglycemia** **component**	fasting glucose ≥ 100 mg/dL or hypoglycemic treatment			fasting glucose ≥100 mg/dL; or glycemia after 2 h OGTT ≥ 140 mg/dL; or HbA1c ≥ 5.7%; or hypoglycemic treatment
**Dyslipidemia** **component(s)**	HDL-C < 50 mg/dL (females) or <40 mg/dL (males); or hyperlipemic therapy			non-HDL-C ≥130 mmol/L; or hyperlipemic therapy
	triglicerydes ≥ 150 mg/dL; or hyperlipemic therapy			
**Requirements for metabolic syndrome diagnosis**	**at least** **3 of any** **5 components**	**mandatory obesity component** **+ at least 2 of any** **4 other components**	**at least** **3 of any** **5 components**	**mandatory obesity component** **+ at least 2 of any** **3 other components**

**Table 2:** Abbreviations for each criterion of metabolic syndromes (2004, 2005, 2009, 2022) and summary.

**Obesity [O] components**	[O1]: waist circumference ≥ 80 cm (females) or ≥94 cm (males)
	[O2]: waist circumference ≥ 88 cm (females) or ≥102 cm (males)
	[O3]: BMI ≥ 30 kg/m^2^
**Hypertension [HT] components**	[HT1]: ≥130/85 mmHg or hypertension treatment
**Hyperglycemia [HG] components**	[HG1]: fasting glucose ≥ 100 mg/dL or hypoglycemic treatment
	[HG2]: HbA1c ≥ 5.7% or hypoglycemic treatment
**Dyslipidemia [D] components**	[D1]: HDL-C < 50 mg/dL (females) or <40 mg/dL (males) or hyperlipemic therapy
	[D2]: triglycerides ≥ 150 mg/dL or hyperlipemic therapy
	[D3]: non-HDL-C ≥ 130 mmol/L or hyperlipemic therapy
**Requirement for metabolic syndrome diagnosis: 2004**	minimum 3 of 5: O2; HT1; HG1; D1; D2
**Requirement for metabolic syndrome diagnosis: 2005**	O1 + minimum 2 of 4: HT1; HG1; D1; D2
**Requirement for metabolic syndrome diagnosis: 2009**	minimum 3 of 5: O1; HT1; HG1; D1; D2
**Requirement for metabolic syndrome diagnosis: 2022**	O2 or O3 + minimum 2 of 3: HT1; HG1 or HG2; D3

**Table 3:** The mean and standard deviation values for laboratory parameters.

Parameter [Units]	Mean ± SD
glucose [mg/dL]	125.46 ± 104.78
HbA1c [%]	6.09 ± 1.36
HDL-C [mg/dL]	53.87 ± 14.47
non-HDL-C [mg/dL]	147.47 ± 46.60
triglicerydes [mg/dL]	135.64 ± 74.87
ALT [U/L]	39.92 ± 27.36
AST [U/L]	32.31 ± 11.69
platelets [×10^9^/L]	256.00 ± 71.03

## Data Availability

The data can be made available upon reasonable request—please contact the correspondence author. The data are not publicly available due to the fact that containing information that could compromise the privacy of research participants.
